# Face masks and speaking style affect audio-visual word recognition and memory of native and non-native speecha)

**DOI:** 10.1121/10.0005191

**Published:** 2021-06-10

**Authors:** Rajka Smiljanic, Sandie Keerstock, Kirsten Meemann, Sarah M. Ransom

**Affiliations:** 1 Department of Linguistics, University of Texas at Austin, 305 East 23rd Street STOP B5100, Austin, Texas 78712, USA; 2 Department of Psychological Sciences, University of Missouri; 124 Psychology Building, Columbia, Missouri 65211, USA

## Abstract

Though necessary, protective mask wearing in response to the COVID-19 pandemic presents communication challenges. The present study examines how signal degradation and loss of visual information due to masks affects intelligibility and memory for native and non-native speech. We also test whether clear speech can alleviate perceptual difficulty for masked speech. One native and one non-native speaker of English recorded video clips in conversational speech without a mask and conversational and clear speech with a mask. Native English listeners watched video clips presented in quiet or mixed with competing speech. The results showed that word recognition and recall of speech produced with a mask can be as accurate as without a mask in optimal listening conditions. Masks affected non-native speech processing at easier noise levels than native speech. Clear speech with a mask significantly improved accuracy in all listening conditions. Speaking clearly, reducing noise, and using surgical masks as well as good signal amplification can help compensate for the loss of intelligibility due to background noise, lack of visual cues, physical distancing, or non-native speech. The findings have implications for communication in classrooms and hospitals where listeners interact with teachers and healthcare providers, oftentimes non-native speakers, through their protective barriers.

## I. INTRODUCTION

**FIG. 1.**
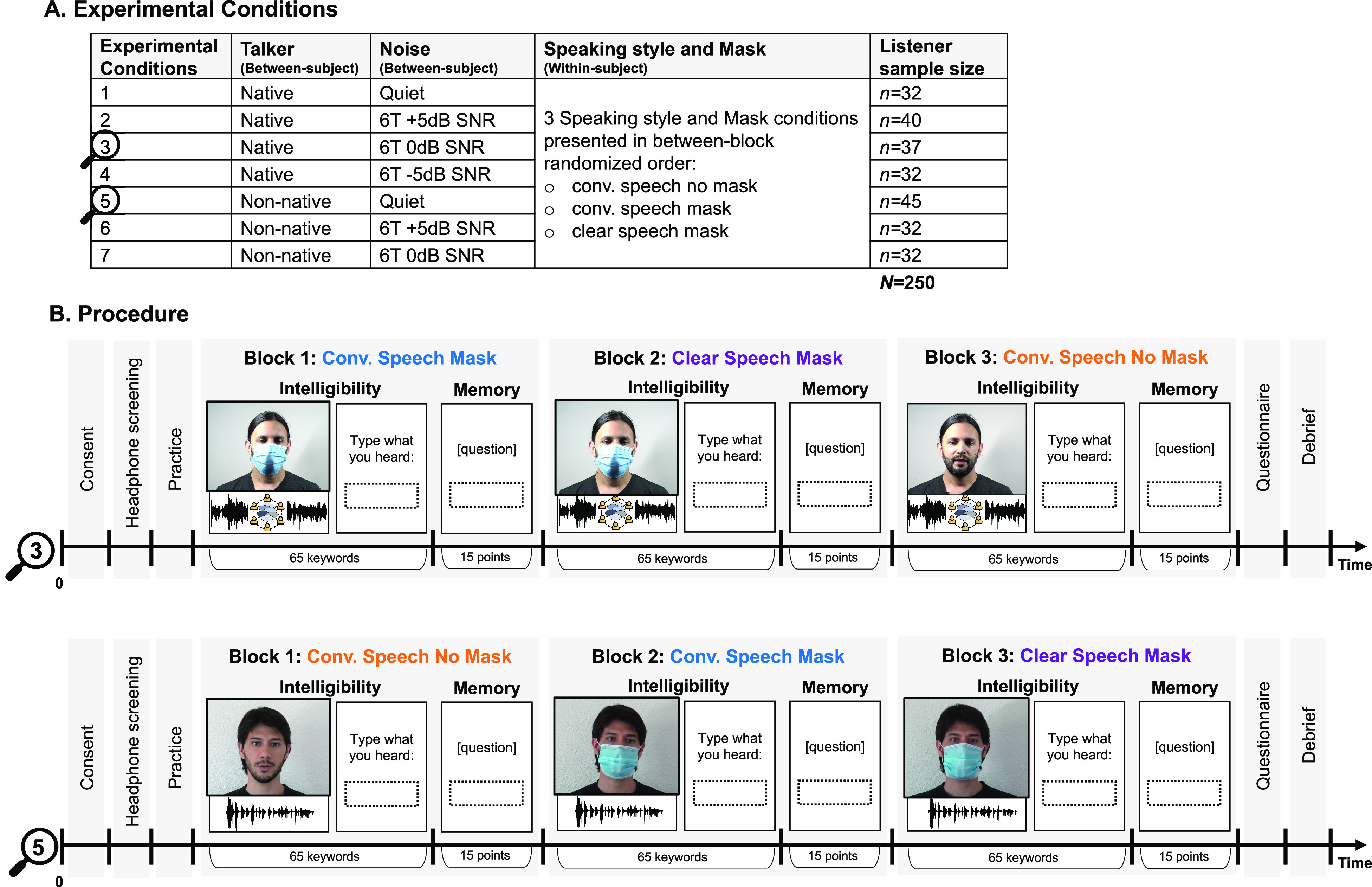
(Color online) Experiment overview. Each participant heard one of the talkers (native talker or non-native) and one noise condition (quiet, +5 dB SNR, and 0 dB SNR; an additional −5 dB SNR was used for the native talker only) (A). Each listener saw video clips from all three speaking style and mask conditions: conversational speech no mask; conversational speech mask, clear speech mask. Speaking style and mask were counterbalanced across listeners (B). Immediately after each audio-video clip, listeners were instructed to type what they heard to test intelligibility. At the end of each block, they were asked to answer content-related questions to test recall.

During everyday communication, the cues available to listeners for understanding speech vary widely. Background noise interferes with access to speech signals, and speech itself varies with respect to how clearly it is produced by speakers. As indicated by United States demographics, everyday communication—including in educational and health settings—is increasingly likely to happen between native and non-native speakers of English who may have less experience communicating in their second language (L2). In addition to this variability within the auditory domain, listeners may or may not have the benefit of being able to see the person they are listening to. Each of these factors plays a significant role in determining how well people understand each other. Here, we examine unique speech processing difficulties arising from the use of protective face masks in combination with these frequently encountered communication challenges. Specifically, we look at word recognition and recall in quiet and in the presence of competing speech for native and non-native accented English produced with and without masks. Additionally, we examine whether a listener-oriented clear speaking style can alleviate some perceptual difficulty for speech produced with protective face masks (see Fig. 1 for a summary of experimental conditions).

The recent global spread of COVID-19 has significantly increased the use of protective face masks beyond health care settings, making speech communication more challenging. Face masks, along with physical distancing, have been recommended for widespread use by the Centers for Disease Control as a means of providing protection from COVID-19 infection (CDC, 2020; Chu *et al.*, 2020). Masks, however, degrade the speech signal by affecting direction of sound radiation and by attenuating frequencies above 1 kHz, which contain crucial information for comprehension, though there is substantial variation between mask types and mic positions (Corey *et al.*, 2020). Masks also obscure visual cues that aid speech recognition by providing supplementary information about speech sounds missing in the auditory signal (Sumby and Pollack, 1954). This becomes particularly relevant as listeners contend with noise, such as competing speech, which is ubiquitous in classrooms, hospitals, and restaurants (Brungart *et al.*, 2001). Speech quality degradation along with obscured visual cues may render speech produced with face masks close to unintelligible for many listeners (Goldin *et al.*, 2020). It is important to understand how protective masks interfere with communication beyond the pandemic context, as face masks are widely used in, for example, military, first responder, and industrial settings.

We are also interested in examining how non-native accented speech produced with and without masks affects intelligibility and recall. Non-native talkers' speech patterns can deviate from the native language norms along numerous dimensions, from the production of the vowel and consonant phonemic contrasts to phonotactics and prosody, which can present a challenge for successful communication (Best and Tyler, 2007; Flege, 1995). Such non-native-accented productions can lead to decreased intelligibility, segmental and lexical ambiguity, and increased processing effort (e.g., Anderson‐Hsieh *et al.*, 1992; Munro and Derwing, 1995). Even when the non-native talkers are highly intelligible, comprehension of accented speech may still require more effort on the part of the listener, as shown with both behavioral and physiological measures (Porretta *et al.*, 2020; Brown *et al.*, 2020; Hazan and Baker, 2011; Van Engen and Peelle, 2014). This difficulty can be further compounded by the use of masks and in background noise.

To counter various communication barriers, talkers can enhance the clarity of the speech signal through listener-oriented speaking style changes. When they are aware that listeners have difficulty understanding them, talkers spontaneously modify their output from hypo- to hyper-articulated speech (H&H theory) (Lindblom, 1990; Perkell *et al.*, 2002; Scarborough and Zellou, 2013; Wassink *et al.*, 2007) to meet the needs of their interlocutor and the listening environment. When the communicated information is transmitted without distortions and is easy to understand, talkers produce conversational, fast, reduced forms of speech. When access to the speech signal is impeded or the signal is degraded in some way, talkers produce hyper-articulated clear speech forms. Clear speech is more intelligible than conversational speech for a wide range of listeners in noisy conditions [for a review of the clear speech research, see Smiljanic and Bradlow (2009); Smiljanic (2021)]. Non-native talkers are also able to produce clear speech. The intelligibility benefit for the listeners, however, is smaller compared to the native talkers' productions, though it increases with higher proficiency (Rogers *et al.*, 2010; Smiljanic and Bradlow, 2011). This is in part due to the non-native talkers' lack of experience in producing the intelligibility-enhancing acoustic-phonetic modifications in the target language.

Clear speech benefit extends to speech processing beyond word recognition; it improves recognition memory and recall for speech in quiet and in noise (Gilbert *et al.*, 2014; Keerstock and Smiljanic, 2018, 2019; Van Engen *et al.*, 2012). The exaggerated acoustic-phonetic clear speech cues seem to enhance memory traces for sentences produced in that style, enabling listeners to retain more information. Recently, Truong *et al.* (2021) found that cued recall was negatively affected when conversational sentences were spoken with a face mask in quiet listening conditions. Cohn *et al.* (2021) showed that word recognition in noise was improved for clear speech sentences produced with a mask in auditory-only domain. Here, we expand on these findings by testing whether native and non-native speech are affected similarly by masks and whether conversational-to-clear speaking style modifications can improve speech communication when masks are used. Specifically, we examine whether clear speech produced with face masks improves word recognition and memory in the audio-visual domain in quiet and in competing speech. Since in-person testing was halted in the University of Texas at Austin Phonetics Lab due to COVID-19, all perception data were collected online (see Sec. II for more details).

One native and one non-native English speaker recorded a short essay about toucans (Chan *et al.*, 2006) designed to resemble what a student may encounter in a classroom setting. They recorded video clips in three conditions: speaking conversationally (i.e., casually) without a mask and then conversationally and clearly with a mask. Native English listeners participated in word recognition and recall tasks for video clips presented either in quiet or mixed with six-talker babble at several levels of difficulty [different signal-to-noise ratios (SNRs)]. We predicted that speech produced with face masks and lack of visual cues would compromise speech intelligibility beyond the impact of noise and non-native accented speech. We further hypothesized that non-native speech would become disproportionately challenging to understand and remember when produced with a mask compared to native speech. Finally, we predicted that clear speech acoustic-phonetic modifications would enhance intelligibility and recall for both talkers even when speaking with a mask. These predictions are in line with the processing models that invoke increased cognitive load and listening effort for degraded or ambiguous speech or for listeners for whom access to the speech signal is impeded [cf. framework for understanding effortful listening (Pichora-Fuller *et al.*, 2016), effortfulness hypothesis (McCoy *et al.*, 2005), and ease of language understanding (Rönnberg *et al.*, 2013)]. In such challenging listening conditions, more cognitive resources are allocated for speech understanding, leaving fewer resources available for information integration, prediction, learning, and memory consolidation (Peelle, 2018; Pichora-Fuller *et al.*, 2016; Rönnberg *et al.*, 2013). In contrast, when speech clarity is enhanced by providing more robust and salient cues (clear speech, visual cues, quiet listening environment), task demands will be decreased, and listening effort will be reduced. Decreased processing load will leave more resources available for downstream task performance facilitating memory of spoken information. The goal of this study was to extend our understanding of the interactions among the various factors that condition successful talker-listener interactions in situations where masked speech is ubiquitous, including during teacher-student and provider-patient communication. The findings will contribute toward a more comprehensive account of the compensatory and cognitive mechanisms that allow listeners to understand and remember speech under a range of communicative situations, including when facial coverings are used.

## II. METHODS

### A. Materials

An educationally relevant essay on the toucan species was modified from Chan *et al.* (2006) to fit the purpose of testing intelligibility and memory in the current study. Forty-five unique sentences were created (e.g., *There are approximately forty Toucan species indigenous to tropical America*). The sentences were divided into three blocks of 15 sentences total, each containing 65 unique keywords. Each sentence contained between 5 and 16 words and between 3 and 6 keywords (underlined) used for word recognition scoring to assess intelligibility. The memory questions in each block were a mix of fill-in-the-blank (between 7 and 9), true/false (1), and cloze questions (between 1 and 2) for a total of 15 possible correct points per block. An example of each type of question is: *The average lifespan of a Toucan is between _________ and ___________ years* (answer: 10; 15); *Toucans' colorful feathers are not a good camouflage in the treetops*. T/F (answer: F); *What is one thing toucans can't do with their beaks?* (answer: construct tree holes). One native (age 36) and one non-native (age 33) male speaker of American English recorded the stimuli. The non-native speaker's first language is Spanish. At the time of the recording, he had resided in the United States for 5 years and was attending graduate school at UT Austin. The age of first exposure was 15, and the self-reported proficiency was 4.25 in L2 and 5 in L1 (1 = low proficiency, 5 = native-like). His daily use of both L1 and L2 was reported to be 5 (1 = no exposure, 5 = constant exposure). He was judged to be highly intelligible and moderately accented by the authors. As we had no access to the lab due to COVID-19, the speakers were recorded in their respective residences, each following the same protocol and using the same equipment. The audio-visual recording was captured using a Sony (Tokyo, Japan) FDR-AX33 Digital 4K camera with individual target sentences presented in sequence on a PowerPoint slide on a computer screen in front of the speaker, and the slides were advanced by the speaker with a mouse click. Audio was recorded at a sampling rate of 48 000 Hz with a wireless dual lavalier microphone, COMICA (Shenzhen, China) CVM-WM100 Plus, attached to the neckline of the talker's shirt. Figure 1 above summarizes the experimental conditions and procedure. It includes a still screenshot from the audio-video clips of the native and non-native talker with and without a mask.

The speakers read all 45 sentences three times in the following order: conversational speech with no mask, conversational speech with mask, clear speech with mask. For the conversational speaking style, the speakers were asked to read sentences in a casual style, as if they were talking to a friend or a family member who is familiar with their voice. For the clear speaking style, they were instructed to read the sentences as if they were communicating with someone who has a low proficiency in English and does not follow them conversationally [following Smiljanic and Bradlow (2005)]. Both speakers wore surgical masks.

The long video recording was processed in Adobe Premiere Pro and Audition for light processing and removal of ambient noise. It was then segmented into individual sentences with a period of silence ranging from 500 to 1000 ms included before and after the target speech. The audio from each sentence was extracted from the video in Quicktime and converted from lossless M4A to WAV format in iTunes. All audio tracks were equalized for the root mean square (rms) amplitude using Praat software (Boersma and Weenink, 2001). The leveled audio was reattached to the corresponding videos in QuickTime. For the experimental noise conditions, leveled audio clips were mixed with six-talker (6T) babble at an “easy” 5 dB SNR and a “hard” 0 dB SNR. An additional −5 dB SNR was used to test intelligibility and memory for the native talker to increase the task difficulty. The SNR levels were determined based on previous work and through pilot data with the current stimuli (e.g., Smiljanic and Bradlow, 2011). We used these SNR levels to get a range of intelligibility scores across speaking style enhancements and mask conditions for the two talkers while avoiding the ceiling and floor performance in any listening conditions.

To verify that talkers produced two different speaking styles, we measured sentence duration excluding pauses. The non-native talker's conversational sentences had an average duration of 4518 ms (range: 2048–6656) with no mask and 4580 ms (range: 2288–6256) with mask, whereas clear speech sentences with mask had a longer mean duration of 5386 ms (range: 2592–7712). The native talker's conversational sentences had an average duration of 3402 ms (range: 1424–4960) with no mask and 3407 ms (range: 1376–4768) with mask, whereas clear speech sentences with mask had a longer mean duration of 6162 ms (range: 2384–8768). The preliminary analyses confirm that the two speakers implemented a typical conversational-to-clear speech enhancement, longer duration of speech intervals (Smiljanic and Bradlow 2009). As expected, the non-native talker's sentence duration for conversational speech was longer than the native talker's (Baese-Berk and Bradlow, 2021). The durations of conversational sentences produced with a mask were only slightly longer than without a mask for the non-native speaker, but there was no difference for the native speaker. Other acoustic properties of the three speaking conditions as well as their impact on word recognition and memory will be investigated in the future in more detail, as this is beyond the scope of the current paper.

### B. Listeners

Listeners were recruited from the University of Texas at Austin Linguistics subject pool and from Prolific, an online participant pool. Participants either received class credit or were paid for their participation. All participants provided informed consent and filled out a language and demographic background questionnaire. A total of 323 participants were recruited. 250 participants were included in the final analysis (mean age = 23.2; age standard deviation = 5; 143 self-identifying as female). They were all native speakers of English residing in the United States between the ages of 18 and 35. They self-reported normal hearing and normal or corrected-to-normal vision. They all passed a validated headphone screening designed for auditory research online (Woods *et al.*, 2017) with a passing rate of at least five of six trials. They reported no technical difficulties, distractions, or modification of the volume on their device during the experiment. Seventy-three participants were excluded because they did not meet the inclusion criteria or did not follow the instructions. The final sample sizes for each condition are included in Fig. 1(A).

### C. Procedure

The experiment was implemented and run on Gorilla (Anwyl-Irvine *et al.*, 2020), an experiment builder and host of online studies. The procedure details are illustrated in Fig. 1(B). Listeners first provided informed consent. Then they were provided with detailed instructions asking them to sit in a quiet room, to eliminate distractions (“switch off phone/email/music”), and to wear a pair of over-the-ear headphones or insert earphones during the entire duration of the experiment. Participants were screened for headphone compliance (Woods *et al.*, 2017). In each trial, participants heard three randomly ordered tones of equal frequency and duration with one tone having a lower amplitude than the other two. One of the louder tones was presented 180° phase-reversed across the stereo channels. This design ensures high accuracy when using headphones and low when listening over loudspeakers due to phase-cancellation. Listeners were asked to choose the tone that was quietest by clicking on a button on the screen. They needed at least five of six correct responses to pass the headphone screening and were given up to two chances. Listeners who passed were directed to the practice, and those who failed both screenings were excluded from the analyses as per participant inclusion criteria (see above). During practice, participants saw two video clips not used in the main task and were asked to type what they heard. As part of the practice, listeners were asked to adjust the volume on their headsets and to keep the volume the same throughout the experiment. The main task consisted of 45 video clips divided into three blocks. After each video clip, participants were instructed to type what they heard in a text box. Each block of 15 clips was followed by 10 questions designed to test memory of the content for a total of 15 points per block. Video clips were always presented in the same order in which the toucan essay was written to ensure natural continuity and progression of the information. No target sentence was presented more than once. Speaking style × mask conditions alternated between blocks, such that each listener saw all three conditions (conversational speech no mask, conversational speech mask, clear speech mask). The order of speaking style × mask was counterbalanced across participants [see examples of one counterbalance order in Fig. 1(B)]. Each participant saw video clips from either the native or non-native talker in one noise condition (quiet or +5 dB SNR or 0 dB SNR; an additional −5 dB SNR was used for the native talker only) [Fig. 1(A)]. At the end, participants filled out a language background questionnaire and a debrief questionnaire in which they were asked about technical problems, distractions, volume adjustments, and compliance with the instructions.

### D. Analysis

#### 1. Intelligibility

Keywords-correct score was calculated for each talker and each speaking style and mask condition. There were between 3 and 6 keywords per sentence (mean: 4.3) and 65 keywords per speaking style × mask condition for a total of 195 keywords per listener. All keywords were unique content words. If words were repeated across the text, we only scored the first occurrence as a keyword to prioritize scoring of novel words and to minimize the facilitatory effect of repetition on accuracy. Scoring was done by automatically comparing listeners' typed responses to the target keywords in R. To optimize string matching, listeners' responses were first manually run through the spelling tool in Microsoft Word to correct for common and obvious spelling errors, as well as to normalize some spellings (e.g., instances of “tucano” and “toucano” were normalized to “tucano”; instances of “treetops” and “tree tops” were normalized to “treetops”). Each keyword received a score of either 1 (correct) or 0 (incorrect or missing). This dichotomous variable was analyzed with a mixed-effect logistic regression using the lme4 package and glmer() function in R (Bates *et al.*, 2015). The lmerTest package (Kuznetsova *et al.*, 2017) was used to obtain *p*-values. The mixed-effect model included talker (two levels: native_[reference]_, non-native), speaking style and mask (three levels: conversational speech no mask_[reference]_, conversational speech mask, clear speech mask), and noise (up to four levels: quiet_[reference]_, 6T + 5 dB SNR, 6T 0 dB SNR, 6T −5 dB SNR) as fixed effects, as well as their interactions. This and all subsequent models included by-listener and by-item intercepts. An additional word recognition accuracy analysis included all function and content words in the sentences. While intelligibility was overall higher, the pattern of accuracy across conditions was the same as when only target keywords were used.

#### 2. Memory

Memory scoring was done by automatically comparing listeners' typed responses to the target responses in R. As was done for intelligibility scoring, listeners' typed responses were first manually run through the spelling tool in Microsoft Word to correct for common and obvious spelling errors as well as to normalize some spellings. Each correct answer received a score of either 1 (correct) or 0 (incorrect or missing). This dichotomous variable was analyzed with a mixed-effect logistic regression using the lme4 package and glmer() function in R (Bates *et al.*, 2015). The mixed-effect model included the same fixed effects and random intercepts as above.

## III. RESULTS

### A. Intelligibility

**FIG. 2.**
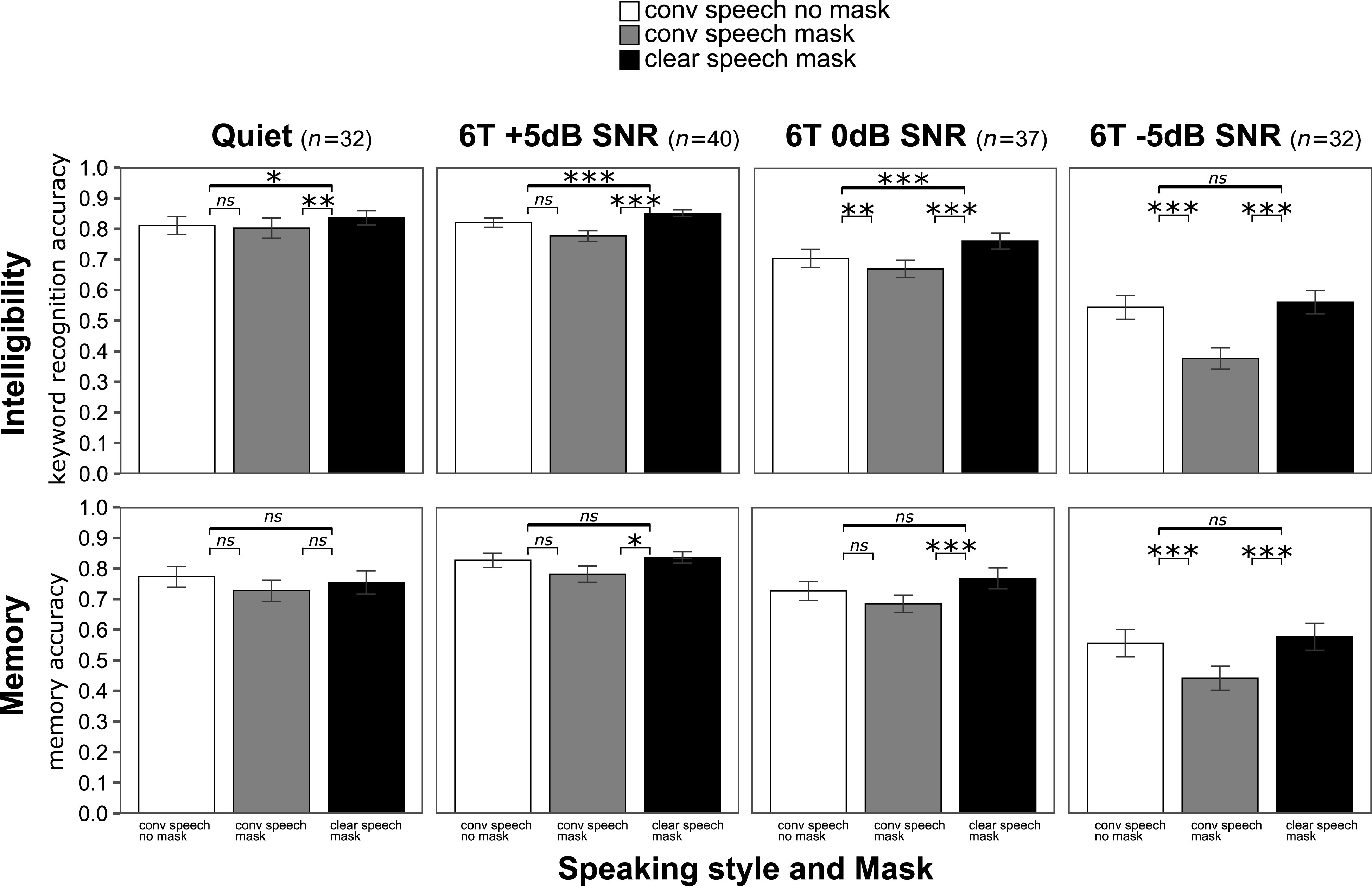
Intelligibility (top panels) and recall (bottom panels) for the native talker in quiet (*n* = 32), in 6T +5 dB SNR (*n* = 40), in 6T 0 dB SNR (*n* = 38), and in 6T −5 dB SNR (*n* = 32). ***, *p* < 0.001; **, *p* < 0.01; *, *p* < 0.05; ns, no statistical difference (mixed-effect logistic regression simple effects analysis).

**FIG. 3.**
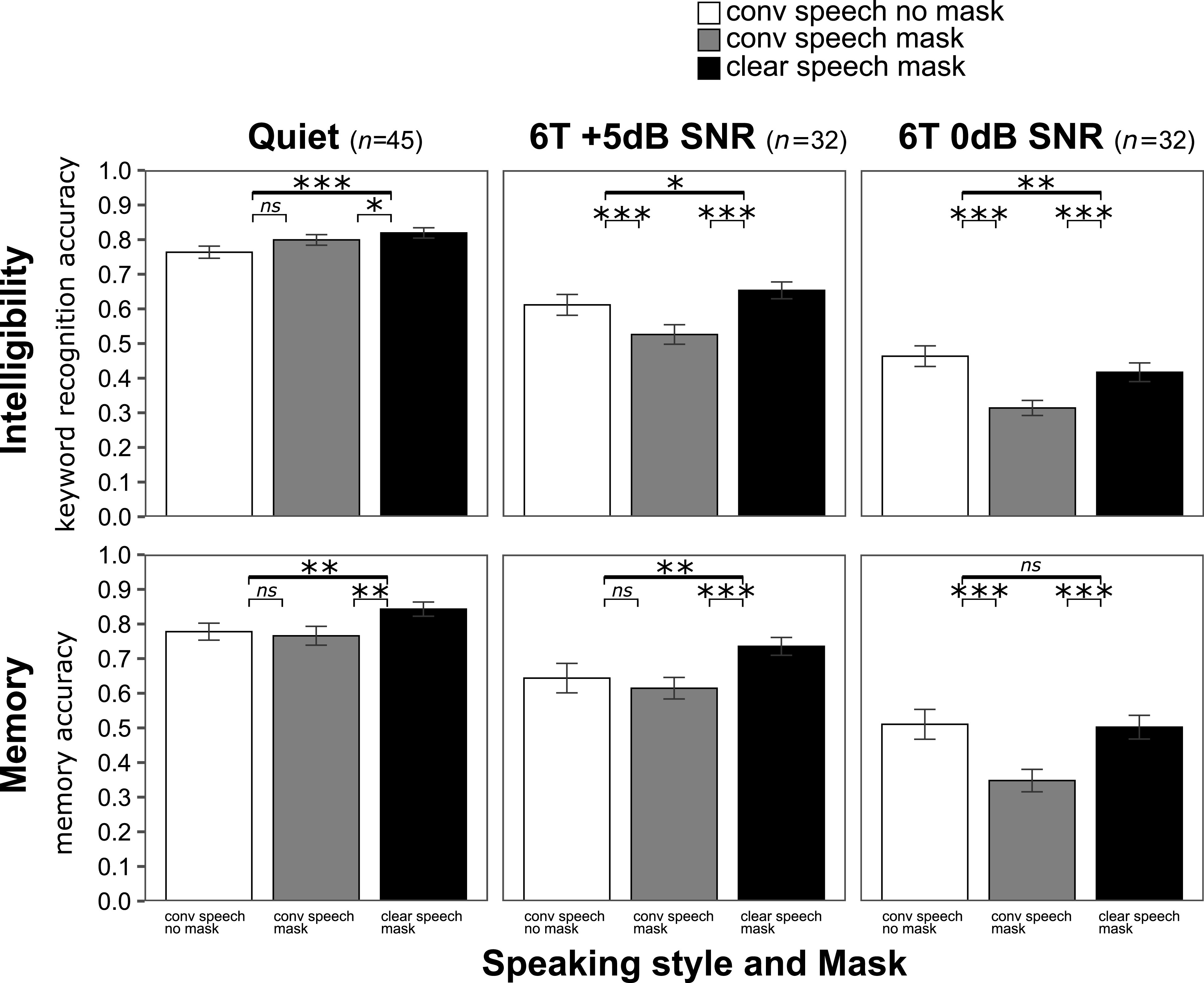
Intelligibility (top panels) and recall (bottom panels) for the non-native talker in quiet (*n* = 45), in 6T +5 dB SNR (*n* = 32), and in 6T 0 dB SNR (*n* = 32). ***, *p* < 0.001; **, *p* < 0.01; *, *p* < 0.05; ns, no statistical difference (mixed-effect logistic regression simple effects analysis).

Figure 2 (native talker) and Fig. 3 (non-native talker) show the overall intelligibility results (top panels) and memory results (bottom panels) in each speaking style × mask and noise (from “easier” on the left to “harder” on the right) conditions.

The results showed that word recognition accuracy was modulated by talker, speaking style, mask, and noise. The mixed-effect model indicated a significant three-way interaction between talker, style, and mask condition and noise (*p* < 0.001). To answer our questions, we decomposed the three-way interaction by talker and noise. For each talker, we found a significant two-way interaction between speaking style and mask condition and noise (*p* < 0.001). The summary is provided in Table I. At every level of noise, including quiet, there were differences across speaking style and mask conditions (see simple effects analyses below). In quiet, word recognition accuracy was high for both the native and non-native talker (no significant effect of talker, *p* = 0.18). The magnitude of the effect of noise on intelligibility was different for the two talkers. Compared to quiet, word recognition accuracy was significantly worse already at +5 dB SNR (*p* < 0.001) for the non-native talker, and it decreased further at 0 dB SNR. In contrast, word recognition at +5 dB SNR for the native talker was not significantly different from quiet (*p* = 0.89). Accuracy was significantly worse at 0 dB compared to +5 dB (*p* < 0.05), and it declined further at −5 dB compared to 0 dB SNR (*p* < 0.001). At each SNR level where direct comparisons were possible (+5 dB and 0 dB), the non-native talker's intelligibility was significantly lower compared to the native talker's intelligibility at the same SNR level (*p* < 0.001).

**TABLE I.** Summary of analysis of deviance table (type III Wald chi square tests).

Model	Term	Chi squared	Df^a^	*p*-value
Overall three-way interaction mixed-effect logistic regression model	StyleMask:Noise:Talker	30.4164	4	<0.001
Simple two-way interaction mixed-effect logistic regression model for native talker	StyleMask:Noise	76.6756	6	<0.001
Simple two-way interaction mixed-effect logistic regression model for non-native talker	StyleMask:Noise	82.053	4	<0.001

^a^ Degrees of freedom (Df).

To closely examine the effect of speaking style and mask on intelligibility, we conducted simple effects analysis within each talker and noise condition (significant effects are indicated with asterisks in Figs. 2 and 3). A summary of the simple effects analyses discussed is provided in Table II. The results for the native talker are provided first. In quiet, the odds of correctly recognizing keywords were significantly greater for clear speech produced with a mask than for both conversational speech with a mask (*p* < 0.01) and without a mask (*p* < 0.05). There was no significant difference between conversational speech produced with a mask and without a mask (*p* = 0.75). Similarly, the odds of correctly recognizing keywords in clear speech with a mask were greater than in both conversational speech with a mask (*p* < 0.001) and without a mask (*p* < 0.001) at +5 dB SNR and 0 dB SNR. There was no significant difference between conversational speech with and without a mask at +5 dB SNR (*p* = 0.53), but a significant difference was found at 0 dB SNR (*p* < 0.01), where keyword accuracy was significantly lower for conversational speech with a mask than without. In the hardest SNR condition (−5 dB), the odds of correctly recognizing keywords were greater in clear speech with a mask than in conversational speech with a mask (*p* < 0.001) and in conversational speech with no mask than in conversational speech with a mask (*p* < 0.001). Keyword accuracy was not different between clear speech with a mask and conversational speech with no mask (*p* = 0.22) at −5 dB SNR.

**TABLE II.** Summary of simple effects. Conv., Conversational speech; Clear, clear speech; ***, *p* < 0.001.

Term	Estimate	Standard error	*z*-value	*p*-value
**Non-native talker in quiet**				
Conv. Mask vs Conv. NoMask	0.11996	0.07024	1.708	0.087670
Clear Mask vs Conv. NoMask	0.27478	0.07137	3.850	0.000118
Clear Mask vs Conv. Mask	0.15482	0.07288	2.124	0.0336
**Non-native talker in 6T +5 dB SNR**				
Conv. Mask vs Conv. NoMask	−0.43171	0.07007	−6.161	7.22E−10
Clear Mask vs Conv. NoMask	0.14067	0.07119	1.976	0.048170
Clear Mask vs Conv. Mask	0.57237	0.06991	8.188	2.67E−16
**Non-native talker in 6T 0 dB SNR**				
Conv. Mask vs Conv. NoMask	−0.78223	0.07177	−10.899	<2E−16
Clear Mask vs Conv. NoMask	−0.20675	0.06916	−2.989	0.0028
Clear Mask vs Conv. Mask	0.57549	0.07166	8.031	9.70E−16
**Native talker in quiet**				
Conv. Mask vs Conv. NoMask	−0.02931	0.09059	−0.324	0.7462
Clear Mask vs Conv. NoMask	0.23478	0.09358	2.509	0.0121
Clear Mask vs Conv. Mask	0.26409	0.0935	2.824	0.00474
**Native talker in 6T +5 dB SNR**				
Conv. Mask vs Conv. NoMask	−0.14581	0.07547	−1.932	0.053352
Clear Mask vs Conv. NoMask	0.28618	0.08044	3.558	0.000374
Clear Mask vs Conv. Mask	0.43198	0.07827	5.519	3.41E−08
**Native talker in 6T 0 dB SNR**				
Conv. Mask vs Conv. NoMask	−0.18207	0.06793	−2.68	0.00735
Clear Mask vs Conv. NoMask	0.35672	0.07074	5.043	4.58E−07
Clear Mask vs Conv. Mask	0.53879	0.07014	7.681	1.58E−14
**Native talker in 6T −5 dB SNR**				
Conv. Mask vs Conv. NoMask	−0.81299	0.07098	−11.454	<2E−16^***^
Clear Mask vs Conv. NoMask	0.08543	0.07008	1.219	0.223
Clear Mask vs Conv. Mask	0.89842	0.07158	12.552	< 2E−16^***^

For the non-native talker in quiet, the odds of correctly recognizing keywords were greater for clear speech produced with a mask than both conversational speech with a mask (*p* < 0.01) and without a mask (*p* < 0.001). There were no significant differences between conversational speech with a mask and without a mask (*p* = 0.09). In both +5 dB and 0 dB SNR, the odds of correctly recognizing keywords were greater for keywords spoken in clear speech with a mask than in conversational speech with a mask (*p* < 0.001) and in conversational speech with no mask than in conversational speech with a mask (*p* < 0.001). At +5 dB SNR, the odds of correctly recognizing keywords were greater for clear speech with a mask than for conversational speech without a mask (*p* < 0.05). At 0 dB SNR, the opposite result was found; the odds of correctly recognizing keywords were greater for conversational speech with no mask than for clear speech with a mask (*p* < 0.01).

### B. Memory

Recall analysis showed that accuracy was modulated by talker, speaking style, mask condition, and noise. The results from the mixed-effect model indicated a significant three-way interaction (*p* < 0.05) as well as significant two-way interactions between speaking style and mask condition and noise for both talkers (*p* < 0.05 for native and *p* < 0.01 for non-native).

For the native talker in quiet, simple effects analysis revealed that speaking style and mask did not significantly affect recall (*p* = 0.75). In +5 dB and in 0 dB, the odds of correctly responding to the memory questions were greater for clear speech produced with a mask than for conversational speech with a mask (*p* < 0.05 and *p* < 0.001 for the two SNRs, respectively). There were no significant differences between clear speech with a mask and conversational speech without a mask and between conversational speech with a mask and without a mask. At the hardest SNR level, −5 dB, the odds of correctly responding to the memory questions were significantly lower for conversational speech with a mask than in clear speech with a mask and conversational speech without a mask (*p* < 0.001), the latter two not being different from each other.

For the non-native talker, the odds of correctly responding to the recall questions were greater for clear speech with a mask than for conversational speech with a mask in quiet and in +5 dB (*p* < 0.01 and *p* < 0.001, respectively). The odds were also greater for clear speech with a mask than for conversational speech without a mask at both SNRs (*p* < 0.01). There was no difference between the conversational speech produced with and without a mask (*p* = 0.32). At 0 dB SNR, the odds of correctly responding to the memory questions were greater for clear speech with a mask than for conversational speech with a mask (*p* < 0.001) and for conversational speech with no mask than with a mask (*p* < 0.001). There was no significant difference between the clear speech produced with a mask and conversational speech without a mask (*p* = 0.72).

## IV. DISCUSSION

In the present study, we tested the effect of protective face masks on word recognition and recall. Listeners saw video clips of talkers with and without masks presented in quiet or in the presence of competing speech from six talkers at various levels of difficulty. Two critical issues were examined: (1) whether native and non-native speech are affected similarly by masks and background noise and (2) whether conversational-to-clear speaking style modifications can improve speech communication when masks are used. Unexpectedly, we found that masks did not negatively impact word recognition and memory for either speaker when presented to listeners in quiet. In optimal listening conditions, without any background noise, speech produced with a mask was as intelligible as speech produced without a mask. In fact, accuracy was at ceiling regardless of whether it was produced with or without a mask. This is in contrast to the Truong *et al.* (2021) study, which found worse cued recall for conversational sentences presented in quiet when a talker wore a mask. This difference could be attributed in part to the mask type and microphone positioning. Talkers in our study used surgical masks, and the speech signal was amplified with a lapel mic positioned in close proximity to the talker's mouth (though still outside of the mask). Talkers in the Truong *et al.* (2021) study wore a cloth mask, and signal was recorded with a stationary mic. Comparisons of filtering properties of various masks and the effect of mic positions on sound intensity showed that surgical masks provide the best acoustic performance and the lapel mics have smallest signal attenuation (Bottalico *et al.*, 2020; Corey *et al.*, 2020; Magee *et al.*, 2020). In addition, Truong *et al.* (2021) used short sentences and a cued-recall task, which could have also contributed to the different results found in our study. The combined findings could be used as guidelines for best practices in teaching environments and in clinics. The findings also highlight the need to further explore how speech comprehension and memory are affected by the various conditions under which speech with masks is produced.

Once listeners had to contend with competing speech, conversational speech produced with a mask became more difficult to understand and remember than conversational speech produced without a mask. However, this effect emerged at different levels of listening difficulty for the two talkers. For the non-native talker, word recognition was significantly lower for conversational speech produced with a mask than without a mask already at the easiest noise level tested, +5 dB SNR, and the difficulty increased as the SNR decreased and the listening conditions became more challenging. In contrast, the effect of the mask on word recognition and memory was not present at +5 dB SNR for the native talker. While the overall accuracy was slightly lower in noise, the performance was still at ceiling, obscuring the effect of the mask on speech recognition. The negative effect started to emerge at the higher level of noise, 0 dB SNR, and increased further in the most difficult listening condition, −5 dB SNR. This showed, as we hypothesized, that non-native speech is affected disproportionately more by the use of masks. Lack of visual cues combined with accented speech presented a more significant challenge for the listeners in the presence of competing speech even at a relatively high SNR. Non-native speech impacted word recognition accuracy as much as a decrease in 5 dB SNR did for the native talker. The results suggest that, in the current conditions, when the use of masks is prevalent, it is important to reduce the background noise, if possible, especially when communicating with non-native speakers.

Instructing talkers to speak clearly while wearing a mask improved speech intelligibility and subsequent ability to recall what was said compared to conversational speaking style produced with a mask in all listening conditions. Both native and non-native talkers were adept at modifying their speech in response to a communicative barrier, in this case a protective face mask and an imagined perceptual difficulty on the part of the listener, and produce intelligibility-enhancing hyper-articulated clear speech forms (H&H theory) (Lindblom, 1990; Perkell *et al.*, 2002; Wassink *et al.*, 2007; Scarborough and Zellou, 2013). In some conditions, word recognition and recall of clear speech produced with a mask were even superior to the conversational speech without a mask. This was true for word recognition in quiet and in noise at +5 dB and 0 dB SNR for the native talker and for word recognition and recall in quiet and in noise at +5 dB SNR for the non-native talker. At −5 dB SNR, for the native talker, clear speech with a mask and conversational speech without a mask improved word recognition recall equally relative to the conversational speech produced with a mask. In this condition, clear speech modifications produced with a mask provided as much processing benefit as the presence of visual cues for the conversational speech. For the non-native talker, the clear speech benefit was smaller than the benefit of visual cues at 0 dB SNR. For both talkers, the clear speech benefit was still significant, though somewhat attenuated, in the most difficult listening conditions we tested.

It can be assumed that intelligibility would be improved to an even greater extent with the benefit of visual clues combined with the clear speaking style (cf. Van Engen *et al.*, 2014; Cho *et al.*, 2020). In addition to the acoustic-phonetic enhancements, clearly produced speech exaggerates the visual cues as well, both of which aid listeners in recovering auditory segmental information lost due to noise masking. The exaggerated visual cues would also help listeners to attend to the correct speaker/auditory stream in the presence of competing speech. Recently, Cohn *et al.* (2021) found improved word recognition in the auditory-only domain for low predictability sentences produced clearly with fabric masks and presented to listeners in four-talker babble at −6 dB SNR. Furthermore, clear speech produced with a mask was more intelligible than clear speech produced without a mask, suggesting that the acoustic-phonetic modifications differed in response to a cloth face mask compared to no mask. It will be important to examine in future work how the availability of the exaggerated visual cues for clearly produced speech would change the pattern of word recognition results. Importantly, evidence is now emerging that a listener-oriented clear speaking style can enhance word recognition and memory under challenging listening conditions even when it is produced with a mask. This work extends the well-documented intelligibility and memory benefit of clear speech to speaking style adaptations produced with a face mask (cf. Smiljanic and Bradlow, 2009; Smiljanic, 2021).

The perceptual benefit of clear speech produced with a mask found here is in line with the models that invoke increased cognitive load and listening effort when access to the speech signal is impeded or speech signal itself is degraded (Pichora-Fuller *et al.*, 2016; McCoy *et al.*, 2005; Rönnberg *et al.*, 2013). Non-native speech and fast conversational speech in combination with additional signal degradation and lack of visual cues due to noise and face masks increased task demands and processing load, affecting word recognition and subsequent recall. More robust acoustic-phonetic cues of clearly produced speech or the presence of visual cues eased some of the processing load during speech perception, leaving more resources for information integration and memory encoding. It is possible, however, that poorer recall for conversational and non-native speech in noise is due entirely to lower intelligibility. That is, if the listeners could not hear the sentences, they would not be able to recall the information. Previous work, though, showed that even highly intelligible conversational sentences were recalled less well compared to clear sentences, suggesting an increased processing cost that is independent of accurate word recognition (Keerstock and Smiljanic, 2018, 2019; Winn and Teece, 2020). Similarly, greater cognitive load indicated by greater pupil dilation was found for an L2 talker compared to an L1 talker even when recognition accuracy was at ceiling for both talkers, as was found here in the quiet condition (McLaughlin and Van Engen, 2020). These results indicate that mismatches between the speech patterns of casual, reduced L1 conversational speech and of L2-accented speech and listeners' phonological and lexical representations require greater recruitment of cognitive resources to achieve the accurate mapping. It is an important direction of future work to test if cognitive load would decrease with less-accented talkers and for the listeners who are familiar with the accent as we found with L1 clear speech (Brown *et al.*, 2020). Regardless of the source of difficulty, the current findings are important because they highlight the need to better understand how various adverse listening conditions affect students in classrooms and patients in hospitals as they remember and recall information.

Finally, it is important to note some limitations of the current study. One aspect in which our study is unique is in the stimuli that we used. We deliberately opted to use an essay rather than sentences or words as used in the studies discussed above (e.g., Truong *et al.*, 2021; Cohn *et al.*, 2021). While we gave up control of context and of word repetition and frequency, we wanted to see how talker characteristics, masks, and speaking styles affect communication in a more naturalistic listening environment, such as when students learn new concepts and unfamiliar content in the classroom setting or when patients need to understand and remember discharge instructions, situations where all of these factors interact to shape comprehension. Similarly, because of the complexity of the paragraph stimuli, the length of the sentences and the time between sentence presentation and memory questions varied somewhat across the three blocks. Finally, our memory questions were a mix of fill-in-the-blank (between seven and nine per block), true/false (one per block), and cloze questions (one to two per block). While these questions tap into somewhat different memory processes, they all involve recall of information in some form. Importantly, these are the types of questions a student could encounter on a quiz after a lesson covering new content.

Even though these study design decisions could have impacted our findings, we believe that our result patterns are meaningful. If anything, the current results may be underestimating some of the effects of speaking style and masks (e.g., some of the ceiling effects we noted) that may be apparent if we used sentence materials devoid of contextual information and word repetition, for example. Note that in our analyses, we only included the first mention of a word as a keyword and not a subsequent repetition of the same word. Furthermore, whatever effect of context or word repetition and frequency or of memory question type there was, it would have affected all mask and speaking style conditions equally. That is, listening conditions were counterbalanced such that each block of 15 sentences was heard in a different combination of speaking style and mask conditions across listeners, and the comparisons were always across speaking style and mask conditions rather than across the blocks. The effect of contextual cues, word repetition and frequency, etc., on speech understanding in situations that resemble daily communication more closely merits further attention and is a fruitful path for future work.

In summary, this paper presents an encouraging finding that speech produced with masks can be as intelligible as speech without masks when surgical masks are used and the signal is amplified with mics positioned in close proximity to the talker's mouth (lapel mic). Speech communication with masks can be enhanced further by simple speaking adjustments in the form of listener-oriented clear speech. Clear speaking style can offset the negative effect of protective face masks on word recognition and recall in noisy environments and when communicating with non-native speakers. These findings have implications for successful communication, especially in challenging listening situations, such as in clinics or classrooms, where listeners have to understand teachers and healthcare providers, oftentimes non-native speakers, through their protective barriers while hearing competing speech streams. It remains to be determined how the surgical mask used in this study attenuated speech signal and how it affected acoustic-articulatory clear speech modifications relative to the conversational speech with and without a mask and the clear speech produced without a mask. It is important also to examine how the change in the articulatory patterns and the quality of the speech signal due to wearing a mask affect intelligibility and memory in the auditory-only domain, which will allow us to tease apart the contribution of the speech signal characteristics from visual cues. Another key issue is that the current study uses a single L1 and L2 talker, so it will be important to verify that these results can be replicated with other L1 talkers and with L2 talkers with different levels of intelligibility and accent types. Finally, the pressing goal is to examine whether these same strategies work to enhance communication for listener groups for whom understanding speech under these challenging conditions remains particularly difficult, including individuals with hearing loss, older adults, and non-native listeners.
